# Impacts of Size and Deformability of β-Lactoglobulin Microgels on the Colloidal Stability and Volatile Flavor Release of Microgel-Stabilized Emulsions

**DOI:** 10.3390/gels4030079

**Published:** 2018-09-15

**Authors:** Ryan W. Murphy, Lijie Zhu, Ganesan Narsimhan, Owen Griffith Jones

**Affiliations:** 1 Whistler Center for Carbohydrate Research, Purdue University, West Lafayette, IN 47907-2009, USA; RyanMurphy271@hotmail.com (R.W.M.); zhu723@purdue.edu (L.Z.); narsimha@purdue.edu (G.N.); 2 College of Food Science and Technology, Bohai University, Jinzhou 121013, China

**Keywords:** β-lactoglobulin, microgel, protein, emulsion, flavor, Pickering, deformability

## Abstract

Emulsions can be prepared from protein microgel particles as an alternative to traditional emulsifiers. Prior experiments have indicated that smaller and more deformable microgels would decrease both the physical destabilization of emulsions and the diffusion-based losses of entrapped volatile molecules. The microgels were prepared from β-lactoglobulin with an average diameter of 150 nm, 231 nm, or 266 nm; large microgels were cross-linked to decrease their deformability. Dilute emulsions of 15–50 μm diameter were prepared with microgels by high shear mixing. Light scattering and microscopy showed that the emulsions prepared with larger, untreated microgels possessed a larger initial droplet size, but were resistant to droplet growth during storage or after acidification, increased ionic strength, and exposure to surfactants. The emulsions prepared with cross-linked microgels emulsions were the least resistant to flocculation, creaming, and shrinkage. All emulsion droplets shrank as limonene was lost during storage, and the inability of microgels to desorb caused droplets to become non-spherical. The microgels were not displaced by Tween 20 but were displaced by excess sodium dodecyl sulfate. Hexanol diffusion and associated shrinkage of pendant droplets was not prevented by any of the microgels, yet the rate of shrinkage was reduced with the largest microgels.

## 1. Introduction

Similar to low molecular weight amphiphilic surfactants, colloidal-scale particles can be used to stabilize emulsions, provided the particles are partially wetted by both the continuous and dispersed phases [1]. One chief advantage of particle-stabilized emulsions is their extreme stability to coalescence due to the formation of thick interfacial layers and the irreversible adsorption of particles to the interface, which prevents sudden changes in the interfacial area associated with volume changes [1]. As long as the contact angle is not close to 0° or 180° (indicating a lack of wettability by one of the phases), the adsorption of particles larger than 10 nm in radius is commonly considered to be irreversible, as the energy required to desorb a particle is greatly in excess of the thermal energy of the particle [2].

The irreversible adsorption of particle-stabilizers to emulsion droplet interfaces has been linked to improved stability against droplet disproportionation and migratory losses of entrapped volatile flavors. Emulsion droplets stabilized by a saturated layer of clay particles were shown to reduce the droplet size changes attributed to Ostwald ripening [3]. Similarly, the ability of particles to reduce the release of entrapped flavor molecules was correlated with the particle density at the interface and inversely correlated with the water solubility of the volatile, indicating that particle-stabilization reduces Ostwald-ripening phenomena [4]. In both cases, the proposed mechanism is the saturated and irreversibly adsorbed layer of particles preventing the shrinkage of the droplet interface. Since volatile loss is linked to droplet volume reduction, volatile molecules cannot migrate from emulsion droplets unless the surface area is allowed to shrink, or the interface can deform to adopt non-spherical shapes. Thus, reducing volatile release is ensured if particles remain adsorbed at the interface and the interfacial tension remains sufficiently high to preclude droplet deformation.

Although the majority of research on particle-stabilizers has focused on rigid particles composed of inorganic compounds [5], there has been increasing interest in the use of soft particulates comprised of biopolymers [6]. Biopolymer-based particles directly address the need for food-grade emulsion stabilizers, where inorganic or synthetically-derived particles are not approved for food use or are facing growing consumer distrust [7,8]. Deformable particles, referred to as microgels, are prepared from polymeric materials by creating a gel-like internal structure with significant internal solvation [9]. These microgels have highly desirable interfacial properties, because they deform at the interface and increase the surface coverage for a given particle loading [10]. As a prominent example, heating β-lactoglobulin (Blg) solutions between pH 5.5 and 6, approximately 1 unit higher than the protein’s isoelectric pH, leads to the formation of microgels with an average diameter of 100–300 nm [11,12]. The size of Blg microgels is increased when heating at pH values closer to pH 5.5, while smaller microgels are formed by heating at higher pH values [13]. The internal structure of Blg microgels is a fractal network of hydrated protein aggregates cross-linked by disulfide and hydrogen bonds [14]. Previous experiments showed that Blg microgels could successfully stabilize coarse oil-in-water emulsions against droplet coalescence [15], and their adsorption rate to droplet interfaces was inversely correlated with microgel size [16].

Based on these prior findings, it was hypothesized that protein microgels of a smaller size and greater deformability would improve the physical stability to coalescence and reduce volatile losses among homogenized flavor-oil emulsions by adsorbing and deforming faster at the droplet interfaces [10,16]. The objectives of this study were then to (i) identify whether deformable Blg microgel particles could reduce the loss of volatile molecules from stabilized, coarse emulsion droplets in comparison to a standard food-industry stabilizer and (ii) to determine how the microgel size and the relative deformability of the microgels would influence emulsion stability and volatile release. The emulsion droplets containing flavor molecules were prepared with the microgels at low oil contents in order to emulate the conditions found in commercial flavor oil emulsions, particularly in low viscosity beverage products.

## 2. Results and Discussion

### 2.1. Microgel Preparation

Microgels were prepared by heating Blg solutions at different pH values (MG_59_, MG_58_, MG_57_), while a portion of MG_58_ were subsequently treated with glutaraldehyde to reduce their deformability (MG_X_). The average hydrodynamic diameters of all resulting Blg microgels were between 150 and 300 nm (Table 1), significantly larger than the hydrodynamic diameter of 2.8 nm for unheated Blg. Increased pH of the solution during heat treatment led to microgels with a significantly reduced particle size so that MG_59_ were the smallest and MG_57_ were the largest, which was consistent with previous experiments [13]. The polydispersity indices for all microgel samples were <0.05, indicating highly monodisperse size distributions that matched previous observations of Blg microgels isolated from residual unaggregated protein [17]. Glutaraldehyde treatment (MG_X_) did not significantly alter the average particle size when compared to the original MG_58_. A similar lack of size change following treatment was noted in a prior study, wherein glutaraldehyde created covalent cross-links within the microgel interior [17]. Transglutaminase treatment was also used in a prior study to create cross-links within microgels, and could be considered as an alternative to glutaraldehyde treatment, yet it was found to be less effective [17].

### 2.2. Emulsion Properties Following Homogenization

Dilute, oil-in-water emulsions were prepared with the isolated microgels as a model for a typical beverage emulsion product. All emulsion samples rapidly separated to form a significant cream layer and a relatively less turbid lower phase quickly after homogenization, yet the cream layers were easily redispersed by vortexing the samples prior to subsequent characterization. Such relative ease in redispersing the cream layer was comparable to prior findings with emulsions stabilized with Blg microgels, where stabilized emulsions were prone to reversible flocculation yet resistant to coalescence [15].

**Figure 1:**
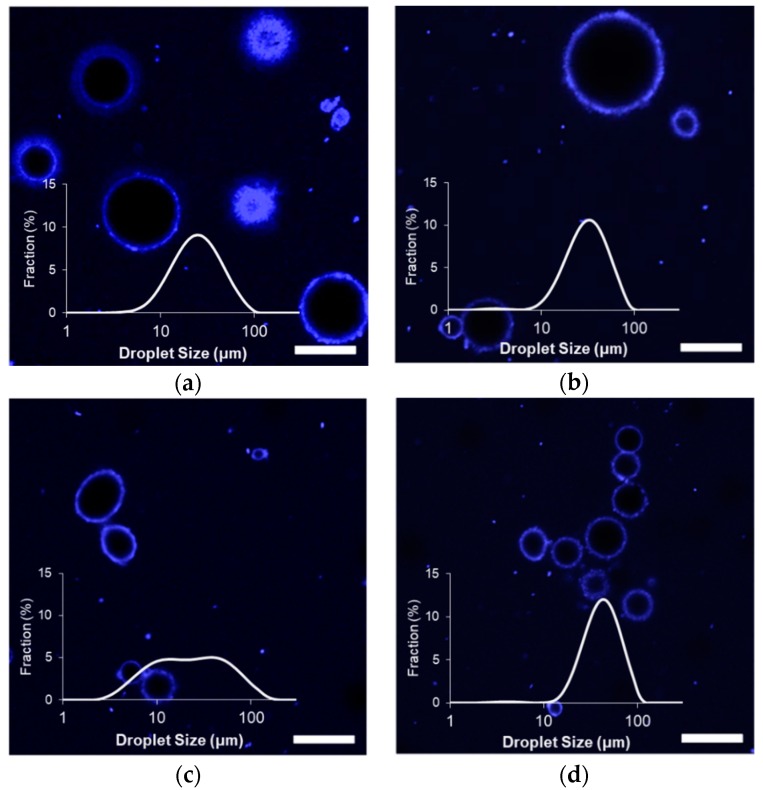
Effect of Blg microgel type on the initial droplet size distribution and morphology of limonene/corn oil-in-water emulsions: (**a**) MG_57_, (**b**) MG_58_, (**c**) MG_59_, (**d**) MG_X_; scale bars = 20 μm.

The size distributions of the stabilized limonene/corn oil droplets fell between 5 and 100 um, with the largest average droplet sizes observed when stabilized with MG_X_ (Figure 1). Apart from the MG_X_-stabilized emulsions, D_43_ values of all microgel-stabilized emulsions were similar to those prepared with the unheated Blg (D_43_ = 34.6 μm), which can be attributed to a limitation in the bench-top homogenizer used for these experiments (Table 2). However, homogenization techniques utilizing greater shear energies may disrupt microgels [15] and were avoided in these tests. Furthermore, similar emulsions prepared with whey protein aggregates and higher intensity emulsification techniques did not achieve droplet sizes any smaller than the D_32_ values observed in this study (Table 2) [18], indicating that the lower intensity homogenization was sufficient. Because large particles require a reduced droplet curvature in order to adsorb effectively at the interface, the minimum stable droplet size must be relatively large, and greater intensity during homogenization would be largely ineffective.

The microgel diameter had a significant impact on the initial droplet sizes of the emulsions (Figure 1 and Table 2). Emulsions prepared with the relatively small MG_59_ possessed the smallest D_32_ of the tested microgel stabilizers and were only slightly larger than emulsions prepared with unheated Blg (D_32_ = 8.4 μm). The relatively broad size distribution of these emulsions indicated that the sub-population of smaller droplets was dominant with a minor fraction of large droplets. Determined D_32_ values were larger when prepared with MG_57_ and MG_58_ and larger still when prepared with MG_X_, indicating that larger stabilizers produced emulsions with a larger average droplet size. Previous findings showed that larger microgels diffuse slower to the interface [16]; this would lead to lower interfacial concentrations directly after homogenization, permitting droplet size growth via coalescence before sufficient microgels could adsorb and stabilize the droplet interface.

Impacts of the different microgels on the initial properties of formed emulsions were further clarified by confocal laser scanning microscopy (CLSM), which are also shown in Figure 1. All emulsion samples possessed a strongly fluorescing interfacial layer, possibly indicating the presence of a thick layer of microgels at the interface to provide steric-stabilization against droplet growth [19]. Images of the emulsions formed with the small MG_59_ confirmed the presence of relatively small diameter droplets, some of which were flocculated within clusters. As no droplets in MG_59_ emulsions were observed with a diameter larger than 25 micrometers, the sub-population of larger objects detected by light scattering could be attributed to these clusters. Images of MG_X_ emulsions showed similar clusters of smaller droplets, and some of the larger particles indicated by light scattering (Table 2) could again be attributed to significant flocculation. The flocculated droplets were not observed among MG_57_ and MG_58_ emulsions; rather, CLSM images showed droplets that were relatively larger than MG_59_ emulsion droplets. It was also possible to see a small population of droplets and protein aggregates of less than a few micrometers in diameter within the background of all samples. These much smaller droplets would not have contributed significantly to light scattering due to their much smaller volume in the suspensions, which would explain their minimal contribution to detected size distributions (Figure 1).

Flocculation among particle-stabilized emulsions has been previously attributed to the simultaneous adsorption of particles to two neighboring droplets following a collision (i.e., bridging) [20]. Such a scenario would occur if the droplet surfaces possessed few adhered particles, so that there would be little chance of particle-particle repulsions during droplet collisions. The concentration of whey protein microgels at the point of contact between droplets has been previously observed among emulsions with low interfacial loads [21]. Coalescence is inhibited after minimal particle adsorption to emulsion droplets [20], and so the large yet unflocculated droplets observed among the MG_58_ and MG_57_ emulsions implied negligible surface coverage by the larger microgels at early times following homogenization. Interestingly, the similarity in initial droplet morphology between MG_X_ and MG_59_ emulsions indicated that MG_X_ also adsorbed quickly to the droplet interfaces despite the microgels being of a comparable size to MG_57_ and MG_58_. It was speculated that glutaraldehyde-treatment, by reducing microgel deformability, facilitated early stabilization of the interfaces by accelerating adsorption (reduced viscous drag in the aqueous phase) or by reducing interfacial spreading.

**Figure 2:**
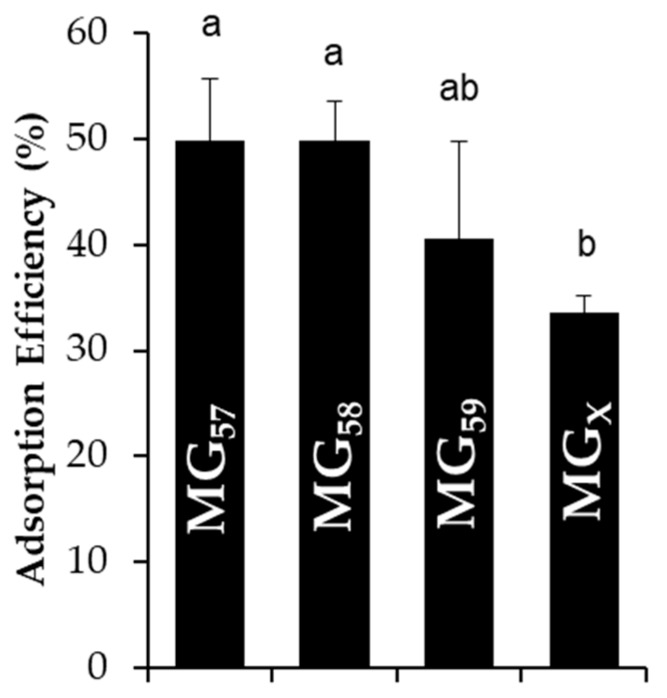
Effect of Blg microgel fabrication pH and cross-linking treatment on efficiency of microgel interfacial adsorption in limonene/corn oil-in-water emulsions. Letters above data denote groups that were not significantly different as a function of treatment.

To estimate the final surface coverage of microgels on stabilized emulsion droplets, the adsorption efficiency was quantified by the content of non-adsorbed microgels remaining in the continuous phase. The adsorption efficiency of all microgel types was approximately 40–60% with only MG_X_ possessing a slightly lower adsorption efficiency in relation to the others (Figure 2). These values were comparable to the observations for emulsions stabilized by whey protein aggregates [18]. The amount of microgels adsorbed to droplet interfaces could also be predicted by considering the total surface area of emulsion droplets derived from light scattering measurements, the number of particles that could pack onto the droplet surfaces (assuming an interfacial contact angle of 45° for whey protein microgels [18]), and the total number of microgels available in the suspensions [15]. Even in a conservative estimate that assumed microgels did not swell/deform at the interface, less than 15% of available microgels would have been required to saturate the droplet interfaces. Since the experimental adsorption efficiency was significantly greater than 15%, there were sufficient microgels to saturate the droplet interfaces and even potentially form multiple, deposited layers, which has been previously observed among the emulsions stabilized by whey protein particles [18]. In such a scenario, the microgels would have first adsorbed to all available droplet interfaces and subsequently deposited as increasingly thick layers, as the interfacial adsorption of microgels is energetically preferred to their aggregation [22].

### 2.3. Effect of Storage on Emulsion Droplet Stability

**Figure 3:**
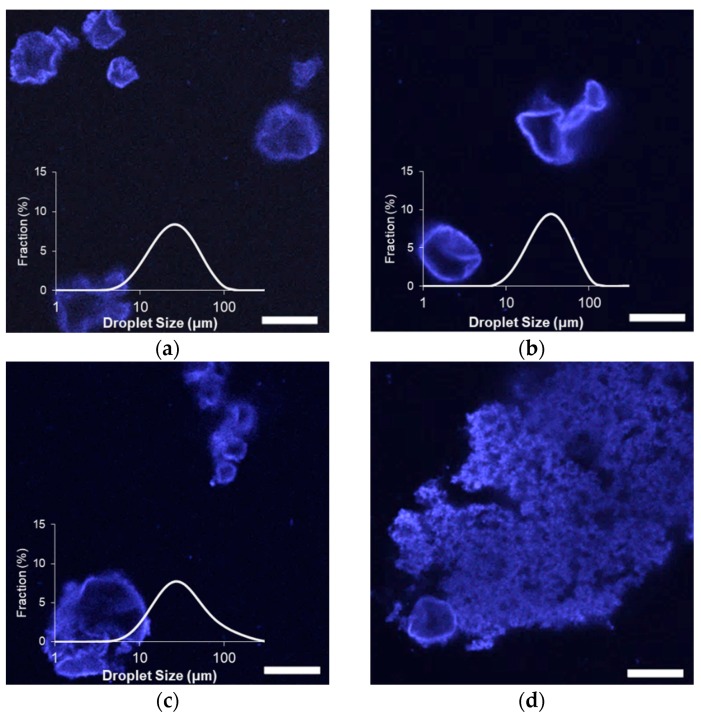
Effect of Blg microgel type on the droplet size distribution and morphology of limonene/corn oil-in-water emulsions after six weeks of storage: (**a**) MG_57_, (**b**) MG_58_, (**c**) MG_59_, (**d**) MG_X_; scale bars = 20 μm; droplet size distribution not shown in (**d**) because MG_X_ emulsions contained large flocs visible to the eye and were not analyzed by light scattering.

After three and six weeks of storage at 25 °C, all emulsion droplets exhibited non-spherical morphologies, attributed to droplet shrinkage that was associated with the gradual loss of limonene (Figure 3 and Figure S1). Limonene, like other terpenes, has a low water solubility with a strong tendency to partition in hydrophobic phases or the air [23]. Any limonene diffusing out into the aqueous phase would then quickly diffuse to the headspace above the emulsions, which represented a significant volume in each sample vessel.

A loss of limonene would be expected for emulsions stabilized by standard surfactants, given the high content of limonene in the dispersed phase, but significant losses were not expected for a particle-stabilized emulsion [4]. Limonene represented 90% of the oil phase volume in the emulsions, and its loss necessitated a reduction in droplet surface area to maintain the spheroidal shape, which is accommodated in traditional emulsions by the desorption of small-molecule surfactants. Particle stabilizers have a significantly greater desorption energy, and so the droplets had to adopt non-spherical shapes in order to reduce volume while maintaining the surface area taken up by the particles. A similar formation of non-spherical droplets has been observed in Pickering emulsions when forced to undergo partial coalescence [24] or when negative pressures were applied to the oil phase [16,25]. This can also occur for droplet interfaces saturated with strongly interactive biopolymers possessing signficant compression and shear elasticity [26].

Despite the partial loss of volume via limonene diffusion among all tested emulsions, light scattering measurements indicated either an increase in size or minimal changes during storage (Figure 3). There were negligible increases in average diameter among MG_57_ and MG_58_ emulsions, to significant increases in size for MG_59_ and MG_X_ emulsions (Table 2). A comparison of the size distributions also showed that the relative fraction of small (<10 μm) droplets in MG_59_ emulsions decreased considerably between zero and six weeks (Figure 1 and Figure 3). The growth in detected size, despite volume losses from limonene loss, could be attributed to an increase in droplet flocculation, which was observed in the microscopy images. Flocculation was so extensive in MG_X_ emulsions that small aggregates were visible to the naked eye.

### 2.4. Effect of pH, Ionic Strength, and Surfactants on Initial Emulsion Droplet Stability

**Figure 4:**
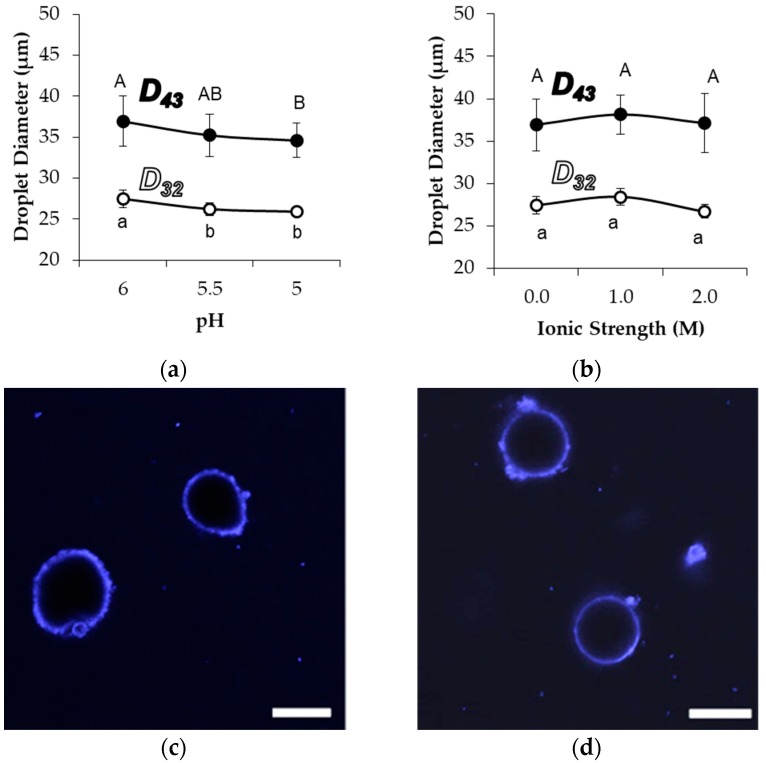
Droplet size and morphology of MG_58_ emulsions during the first day of storage as influenced by acidification and increased sodium chloride concentration, showing moment-based average droplet diameters with exposure to (**a**) decreasing pH or (**b**) increasing ionic strength, as well as confocal micrographs of droplets after adjustment of the solution to (**c**) pH 5.0, 0 M ionic strength or (**d**) pH 6.0, 2.0 M ionic strength. Uppercase/lowercase letters in (**a**,**b**) denote groups of D_43_/D_32_ data that were significantly different as a function of pH or ionic strength; scale bars = 20 μm.

The above tests demonstrated that emulsions prepared with MG_58_ were relatively stable during storage when compared to those prepared with smaller or cross-linked microgels. To further test the stabilizing capabilities of MG_58_, droplet size and morphology were observed after exposure to acid, increased ion concentration, or common surfactants. The acidification of emulsions to pH 5.0, as well as an increase in the sodium chloride concentration up to 2.0 M, were expected to diminish effective electrostatic repulsions among the stabilized droplets and cause destabilization based on prior observations of aggregation stability among Blg microgels [17,27], yet the emulsion droplet size did not increase (Figure 4). The average detected droplet diameters did not increase with increased ionic strength, while acidification actually resulted in a small but significant decrease in the average diameter of detected droplets during light scattering. There was no associated change in the droplet size distribution about the mean, indicating that the entire population of droplets was similarly affected by the pH change (not shown). Further, CLSM images did not indicate any major changes in the interfacial coverage or aggregation of droplets under different pH or ionic strength conditions (Figure 4c,d). However, a limited level of non-sphericity was observed with samples at pH 5.0, implying an accelerated droplet shrinkage and limonene migration. It is plausible that such accelerated shrinkage was caused by an increased pressure exerted on the droplet interface because of the tendency for compaction within and among microgels at the lower pH.

**Figure 5:**
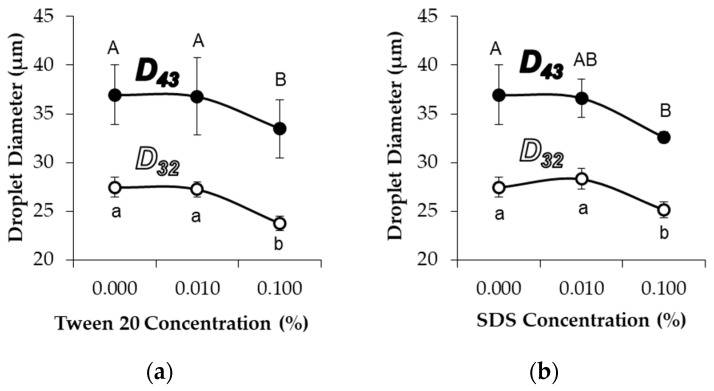
Effect of Tween 20 (**a**) and SDS (**b**) addition on moment-based mean droplet diameters of MG_58_ emulsions. Uppercase/lowercase letters above/below datapoints denote groups of D_43_/D_32_ data that were not significantly different as a function of surfactant concentration.

**Figure 6:**
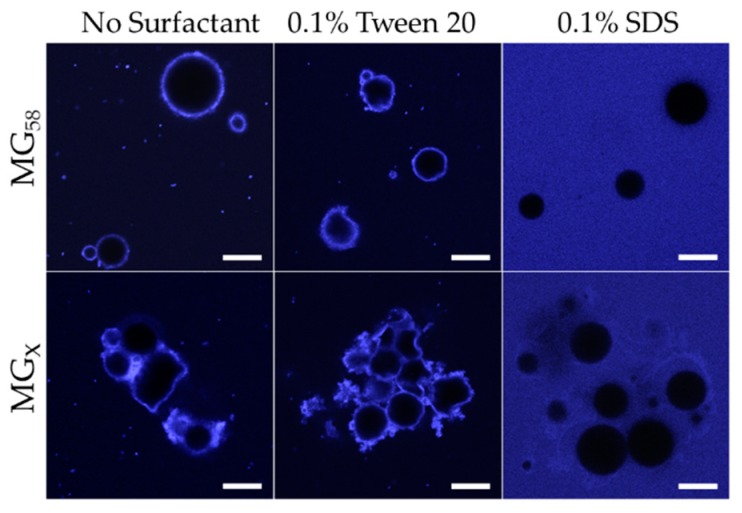
Confocal micrographs of MG_58_ or MG_X_ emulsions in the presence of Tween 20 and SDS surfactants; scale bars = 20 μm.

The addition of either 0.1% Tween 20 (non-ionic surfactant) or 0.1% SDS (anionic surfactant) significantly decreased the average diameters of MG_58_ emulsion droplets (Figure 5). As with pH adjustment, the droplet size distribution indicated a general shift in the droplet size, as opposed to the formation of a separate population of smaller droplets (not shown). CLSM images showed that the addition of 0.1% Tween 20 to either MG_58_ emulsions or MG_X_ emulsions caused the droplets to become non-spherical, but the strong fluorescence at the interface indicated that the quantity of protein in the interfacial layer was not substantially reduced (Figure 6). The extent of non-sphericity observed was significantly greater when compared to the emulsions without added surfactant during early storage, including those adjusted to pH 5.0. In contrast, the addition of 0.1% SDS appeared to fully displace microgels from the interface, resulting in smaller, highly spherical droplets (Figure 6). Among MGX emulsions, SDS was less effective at disrupting flocculated clusters.

The displacement of microgels at the interface or faster adoption of non-spherical morphologies could both be attributed to the decreased interfacial tensions associated with surfactant adsorption. In the case of Tween 20, adsorption of surfactant between microgels at the interface lowered the interfacial tension and reduced the energy for interfacial deformation. Unlike Tween 20, adsorption of SDS at the bare spots of the interface was able to supplant the adsorbed microgels, and the resulting SDS-stabilized droplets subsequently behaved as a typical flavor emulsion system with a reversibly adsorbing/desorbing surfactant. The persistence of flocculated droplets in MG_X_ emulsions after the addition of SDS could be attributed to the inability of SDS to remove the cross-linked microgels that were bridging between two neighboring droplets (Figure 6, lower right). In all scenarios, the speculated outcome for surfactant addition, based on the observed acceleration of interfacial deformation or microgel displacement, would be an increased loss rate of limonene.

### 2.5. Flavor Release

**Figure 7:**
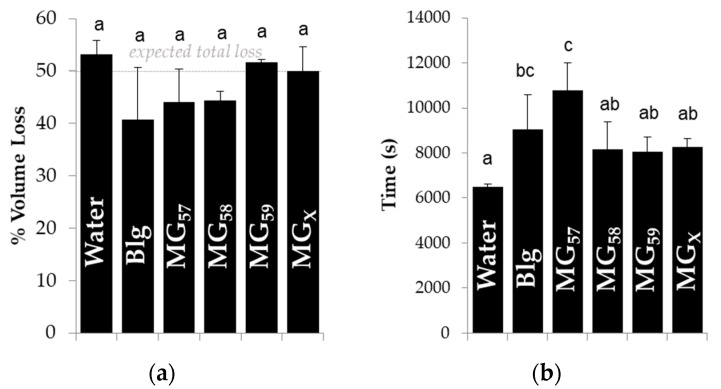
Effect of Blg microgel size and cross-linking treatment on (**a**) total volume loss from a pendant droplet containing 50% (*v*/*v*) 1-hexanol and (**b**) time to achieve total volume loss. Letters above data denote groups of data that were not significantly different as a function of different treatment.

In order to determine the capacity of adsorbed microgels to diminish the release of flavor compounds, the volume of pendant droplets containing the volatile molecule hexanol (50% *v*/*v*) was monitored over time with or without stabilizing microgels (Figure S2), and the ability of the microgels to prevent droplet shrinkage or slow the rate of shrinkage is described in Figure 7. Droplet interfacial tension decreased initially (not shown) in a manner similar to previous droplet tensiometry measurements with Blg microgels [16], which confirmed the adsorption of microgels to the oil-water interface. None of the tested stabilizers significantly reduced the total volume loss (Figure 7a), which reflected the strong affinity of hexanol for the aqueous phase and the inability of microgels or protein to impede the migration of entrapped volatiles from the droplets, as already described for bulk emulsions after storage in Figure 3. When compared to bare interfaces (pure water) or other microgels, the larger MG_57_ significantly increased the time required for droplets to shrink (Figure 7b), indicating that these larger microgels at the interface inhibited flavor migration. The smaller MG_58_, MG_59_, or MG_X_ did not significantly increase the time for shrinkage, yet unheated Blg did increase the time for shrinkage and was not statistically different from MG_57_.

Larger microgels were expected to form a thicker overall interfacial layer, which may have increased the average path length required for the hexanol molecules to diffuse away from the droplet, and also increased the time for complete hexanol release. A lesser effect was expected for the droplets stabilized by MG_58_ and MG_59_, but any such difference may have been below the detection threshold of this experiment. Strong evidence has linked changes in droplet size during shrinkage to the initial droplet size [26], but this factor was discounted because the initial volume was controlled in these tests. Droplet shrinkage and associated buckling have also been linked to the ratio of interfacial thickness to initial droplet size [28], which would again point to the thicker interfacial layer of larger MG_57_ as the cause of a slowed shrinkage rate. The long release times observed with Blg-stabilized droplets could be attributed to the greater capacity of individual Blg molecules to adsorb, unfold, and spread at the interface, giving an advantage over the larger microgels at earlier shrinkage times. Future studies require an improved experimental technique that allows measurement of volume losses from droplets with a fully saturated interface.

## 3. Conclusions

Despite providing resistance to coalescence over six weeks of storage, limonene-containing emulsions stabilized by Blg microgels were susceptible to creaming, flocculation, and limonene loss during storage. In contrast with findings from both synthetic [10] and biopolymer systems [16], the findings refuted the hypothesis that faster adsorption of particles to droplet interfaces translates to improved emulsion stability during storage. Furthermore, regardless of the adsorption rate of microgels or the flocculated state of the droplets, none of the microgel-stabilized emulsions arrested the release of volatile flavor compounds hexanol or limonene from either pendant droplets or the dispersed emulsion droplets, respectively. Since volatile release did not coincide with microgel desorption, this study confirmed that particles, including microgels, are very difficult to desorb from the interface, while also demonstrating that the energy cost associated with the increased surface area-to-volume ratio of a non-spherical droplet was not a sufficient barrier against volatile release. The measurements of the microgels in relation to unheated Blg, combined with other investigations [26], indicate that assembling a strongly interactive, viscoelastic interfacial layer from smaller biopolymer components would be a more efficient approach to impact the process of volatile release from emulsion droplets.

Future studies should validate these results using a technique to craft microgel-stabilized emulsions where the microgels are allowed to adsorb before significant loss of volatiles (such as with membrane emulsification or a microfluidic approach). A potential difference in the internal structure of MG_57_ leading to a difference in total shrinkage time for droplets could also be further investigated in later studies by observing the dynamic diffusion of volatiles through a model interfacial film of these different microgels. Meanwhile, limited physical stability of the emulsions combined with their desorption or increased destabilization in the presence of surfactants SDS or Tween 20 demonstrated that Blg microgels would be best employed as supplementary stabilizers in high viscosity food systems where coalescence is the primary driver of emulsion destabilization.

## 4. Materials and Methods

### 4.1. Materials

β-lactoglobulin (Blg, lot# JE 001-0-415), with a reported composition of 97.9% protein (91.5% Blg), 0.2% fat, 1.8% ash, and 4.4% moisture, was donated by Davisco Food International (Le Sueur, MN, USA). Blg was further purified of non-native protein and ions using a previously published protocol [29]. Glutaraldehyde, Fast Green FCF, d-limonene, sodium azide, hexanol and hexadecane were obtained from Sigma Chemical Co. (St. Louis, MO, USA). Sodium dodecyl sulfate (SDS) and Tween 20 were obtained from Fisher Scientific (Asheville, NC, USA). All other chemicals used were of analytical grade unless stated otherwise. All solutions were prepared using ultrapure water (σ ≥ 18 mΩ-cm) obtained from a filtration system (Barnstead E-pure, Thermo Scientific, Waltham, MA, USA).

### 4.2. Microgel Preparation

4% (*w*/*w*) Blg was mixed thoroughly in ultrapure water and acidified to pH 5.70, 5.80 or 5.90 using 0.1 M hydrochloric acid. Microgels were formed by submerging 16 × 100 mm glass tubes filled with 12 mL Blg solution in a hot water bath at 85 °C for 20 min directly followed by submersion in an ice-water bath for 20 min [13]. Microgel crosslinking was performed based on a previous study [17], wherein glutaraldehyde was added to microgel suspensions prepared at pH 5.80 at a 5:1 glutaraldehyde:protein molar ratio and incubated at 37 °C for 3 h. Resulting microgel suspensions formed at pH 5.9 (MG_59_), at pH 5.8 (MG_58_), at pH 5.7 (MG_57_), and cross-linked with glutaraldehyde (MG_X_) were diluted 3× with ultrapure water and dialyzed against ultrapure water (MWCO = 1000 kDa) to remove free protein. Dialysis in combination with 8× dilution using sodium acetate buffer (2.5 mmol kg^−1^ at pH 6.0) followed by centrifugation at 9300 *g* for 20 min was expected to remove small (~10 nm) aggregates present in the samples, based upon prior findings [16]. The isolated pellet was resuspended in a volume of water equivalent to the initial state by sonicating for 5 s (Branson 3510 Ultrasonic Cleaner, Branson Ultrasonics, Danbury, CT, USA) and vortexing for 30 s. Large aggregates were removed by centrifuging at 1000 *g* for 20 min to obtain the final microgel suspension in the supernatant. Final microgel concentration was determined by gravimetric drying, wherein a known volume of microgel suspension was dried at 105 °C and the weight fraction of microgels in the suspension was determined from the remaining mass. Samples were subsequently diluted with sodium acetate buffer to reach the desired concentrations. Sodium azide (final concentration 0.10 mg mL^−1^) was added with the buffer to prevent microbial growth.

### 4.3. Microgel Characterization

Microgel hydrodynamic size was determined by dynamic light scattering with a CGS-3 compact goniometer system (ALV, Langen, Germany) at 25 °C. Samples were diluted 10× with buffer in order to eliminate concentration-dependent scattering effects. Initial measurements performed at angles of 35–120° did not indicate significant angular dependence on observed mean hydrodynamic sizes, and subsequent measurements were taken at 90°. Size distributions were determined from the correlation function by use of the CONTIN algorithm. Reported microgel radii were obtained from the Gaussian average of the population representing >95% of the total. Polydispersity index was determined from the correlation function using Cumulants analysis. No differences between sizes determined by CONTIN and Cumulants analysis were noted.

### 4.4. Emulsion Preparation

Dilute, beverage-relevant emulsions were prepared with microgels based upon an established approach [18]. In brief, 2% (*v*/*v*) of oil phase containing 90% d-limonene and 10% corn oil was dispersed in suspensions of Blg microgels or Blg (0.1% *w*/*v*) and subsequently homogenized with a Ultra-Turrax T-25 homogenizer (IKA, Staufen, Germany) at 6500 rpm for 2 min. Emulsions were then vortexed for 2 h, which was shown in a prior investigation to allow interfacial adsorption of whey protein-based microgels [30]. Emulsions (35 mL/sample) were stored at 25 °C in 50 mL plastic tubes and covered with plastic caps.

### 4.5. Emulsion Characterization

Emulsion droplet size distributions were characterized using a multi-angle laser light scattering instrument (Mastersizer 2000E, Malvern Instruments, Worcestershire, UK). Particle size distributions of samples were obtained from software-supplied fitting algorithms using a dispersed-phase refractive index of 1.473 and a continuous-phase refractive index of 1.330. Reported surface- and volume-weighted mean diameters (D_32_ and D_43_, respectively) of the samples were determined from the particle size distributions. To determine relative stability of emulsions to pH changes, ionic strength changes, or competition with surfactants, solutions of 0.1 M hydrochloric acid, 0.5 M sodium chloride, or 0.5% (*w*/*v*) Tween 20 or SDS were added to the samples, respectively, and allowed to equilibrate for at least 30 min prior to evaluation.

### 4.6. Adsorption Efficiency

Microgel adsorption efficiency was measured by centrifuging emulsion samples 10 min at 1000× *g* to induce creaming. Creamed emulsion layer was removed, and the quantity of residual protein in the remaining subnatant was measured by gravimetric drying. The adsorption efficiency was calculated as the ratio of the protein adsorbed to the interface and the protein used in the initial emulsion preparation.

### 4.7. Confocal Laser Scanning Microscopy

Microstructure of Blg microgel stabilized emulsions was examined using a Nikon A1Rsi confocal microscope (Nikon, Tokyo, Japan). 50 μL of sample were mixed with 15 μL of 1 mg mL^−1^ Fast Green FCF dye to visualize protein and 35 μL 0.5% xanthan gum (final concentration 0.175%) to reduce sample movement, based on a similar study with whey protein microgel stabilized emulsions [30]. Samples were left at room temperature for 30 min to allow the Fast Green FCF to associate with the Blg microgels. 10 μL of the emulsion, dye and gum mixture were deposited on a glass slide, and covered with a #1.5 glass cover slip using two strips of double-sided tape as spacers. Fast Green FCF was excited using a He-Ne laser (640 nm) and imaged using a 60× oil immersion objective lens.

### 4.8. Flavor Release

Droplets consisting of 1:1 hexanol and hexadecane (*v*/*v*) with an approximate volume of 4.5 μL were formed in aqueous suspensions with 0.1% *w*/*w* microgels or 0.1% *w*/*w* Blg. The change in droplet volume over time was measured using a KRUSS DSA30b (KRUSS GmbH, Hamburg, Germany) with an inverted pendant drop setup. Droplet dimensions were determined every second for up to four hours. The time taken for the droplet to reach a constant volume and the final droplet volume were recorded as indicators of flavor release rate and flavor retention, respectively.

### 4.9. Statistical Treatment

All experiments were performed in triplicate and reported as the mean and standard deviation, unless otherwise stated. Significance testing was accomplished using Tukey’s Honest Significant Difference test for multiple comparisons.

## Figures and Tables

**Table 1:** Average hydrodynamic diameter and polydispersity index (PDI) of Blg microgel suspensions prepared at different pH values with and without crosslinking with glutaraldehyde.

Microgel	^1^ Hydrodynamic Diameter (nm)	PDI
MG_57_	266 ± 7.91 ^a^	0.0368
MG_58_	231 ± 7.11 ^b^	0.0195
MG_59_	150 ± 2.47 ^c^	0.0327
MG_X_	228 ± 12.0 ^b^	0.0233

^1^ Superscript letters in this column denote groups of data that were significantly different.

**Table 2:** Effect of Blg microgel fabrication pH and cross-linking treatment on the moment-based average diameters of limonene/corn oil-in-water emulsions after initial preparation (zero weeks) and after six weeks of storage.

Microgel Used	D_32_ (μm) ^1^		D_43_ (μm) ^1^	
	Zero Weeks	Six Weeks	Zero Weeks	Six Weeks
MG_57_	21.8 ± 1.52 ^cd^	21.9 ± 2.37 ^cd^	30.4 ± 3.27 ^d^	32.0 ± 5.05 ^cd^
MG_58_	27.6 ± 2.19 ^bc^	30.5 ± 2.50 ^b^	36.8 ± 3.97 ^bcd^	41.7 ± 4.14 ^abc^
MG_59_	16.0 ± 2.32 ^d^	25.9 ± 2.05 ^bc^	34.4 ± 1.62 ^cd^	44.7 ± 2.60 ^ab^
MG_X_	38.7 ± 5.58 ^a^	N/A ^2^	48.2 ± 2.99 ^a^	N/A ^2^

^1^ Superscript letters in the columns denote groups of D_32_ or D_43_ data that were significantly different as a function of treatment or weeks of storage; ^2^ MG_X_ emulsions at six weeks possessed visible aggregates, so droplet size analysis was not performed.

## Supplementary Materials

The following are available online at http://www.mdpi.com/2310-2861/4/3/79/s1, Figure S1: Confocal Laser Scanning Micrographs of limonene/corn oil-in-water emulsions stabilized by Blg microgels after 3 weeks of storage, Figure S2: Dynamic volumetric changes in pendant droplets during release of 1-hexanol in MG_57_ suspensions in terms of (A) volume of the pendant droplet or (B) volume loss of pendant droplet normalized by the droplet interfacial area and the total volume loss at long times.

## References

[1] Pickering S.U. (1907). Emulsion. J. Chem. Soc..

[2] Dickinson E. (2012). Use of nanoparticle and microparticles in the formation and stabilization of food emulsions. Trends Food Sci. Technol..

[3] Ashby N., Binks B. (2000). Pickering emulsions stabilised by laponite clay particles. Phys. Chem. Chem. Phys..

[4] Binks B., Fletcher P., Holt B., Beaussoubre P., Wong K. (2010). Selective retardation of perfume oil evaporation from oil-in-water emulsions stabilized by either surfactant or nanoparticles. Langmuir.

[5] Aveyard R., Binks B.P., Clint J.H. (2003). Emulsions stabilized solely by colloidal particles. Adv. Colloid Interface Sci..

[6] Lam S., Velikov K.P., Velev O.D. (2014). Pickering stabilization of foams and emulsions with particles of biological origin. Curr. Opin. Colloid Interface Sci..

[7] Brom F.W. (2000). Food, consumer concerns, and trust: Food ethics for a globalizing market. J. Agric. Environ. Ethics.

[8] Eden S., Bear C., Walker G. (2008). The sceptical consumer? Exploring views about food assurance. Food Policy.

[9] Oh J.K., Lee D.I., Park J.M. (2009). Biopolymer-based microgels/nanogels for drug delivery applications. Prog. Polym. Sci..

[10] Destribates M., Eyharts M., Lapeyre V., Sellier E., Varga I., Ravaine V., Schmitt V. (2014). Impact of PNIPAM microgel size on its ability to stabilize pickering emulsions. Langmuir.

[11] Donato L., Schmitt C., Bovetto L., Rouvet M. (2009). Mechanism of formation of stable heat-induced beta-lactoglobulin microgels. Int. Dairy J..

[12] Jung J., Savin G., Pouzot M., Schmitt C., Mezzenga R. (2008). Structure of heat-induced beta-lactoglobulin aggregates and their complexes with sodium-dodecyl sulfate. Biomacromolecules.

[13] Murphy R., Cho Y., Farkas B., Jones O. (2015). Control of thermal fabrication and size of b-lactoglobulin-based microgels and their potential applications. J. Colloid Interface Sci..

[14] Schmitt C., Moitzi C., Bovay C., Rouvet M., Bovetto L., Donato L., Leser M.E., Schurtenberger P., Stradner A. (2010). Internal structure and colloidal behaviour of covalent whey protein microgels obtained by heat treatment. Soft Matter.

[15] Zimmerer L., Jones O. (2014). Emulsification capacity of microgels assembled from β-lactoglobulin and pectin. Food Biophys..

[16] Murphy R., Farkas B., Jones O. (2016). Dynamic and viscoelastic behavior of β-lactoglobulin microgels of varying sizes at fluid interfaces. J. Colloid Interface Sci..

[17] Murphy R., Farkas B., Jones O. (2017). Effect of crosslinking on the physical and chemical properties of β-lactoglobulin (BLG) microgels. J. Colloid Interface Sci..

[18] Sarkar A., Murray B., Holmes M., Ettelaie R., Abdalla A., Yang X. (2016). In vitro digestion of pickering emulsions stabilized by soft whey protein microgel particles: Influence of thermal treatment. Soft Matter.

[19] Horozov T.S., Binks B.P. (2006). Particle-stabilized emulsions: A bilayer or a bridging monolayer?. Angew. Chem..

[20] Vignati E., Piazza R., Lockhart T.P. (2003). Pickering emulsions:  Interfacial tension, colloidal layer morphology, and trapped-particle motion. Langmuir.

[21] Destribates M., Rouvet M., Gehin-Delval C., Schmitt C., Binks B. (2014). Emulsions stabilized by whey protein microgel particles: Towards food-grade pickering emulsions. Soft Matter.

[22] Zielinska K., Campbell R.A., Zarbakhsh A., Resmini M. (2017). Adsorption versus aggregation of nipam nanogels: New insight into their behaviour at the air/water interface as a function of concentration. Phys. Chem. Chem. Phys..

[23] Copolovici L.O., Niinemets Ü. (2005). Temperature dependencies of henry’s law constants and octanol/water partition coefficients for key plant volatile monoterpenoids. Chemosphere.

[24] Pawar A., Caggioni M., Ergun R., Hartel R., Spicer P. (2011). Arrested coalescence in pickering emulsions. Soft Matter.

[25] Buchcic C., Tromp R., Meinders M., Stuart M. (2017). Harnessing the advantages of hard and soft colloids by the use of core–shell particles as interfacial stabilizers. Soft Matter.

[26] Erni P., Jerri H.A., Wong K., Parker A. (2012). Interfacial viscoelasticity controls buckling, wrinkling and arrest in emulsion drops undergoing mass transfer. Soft Matter.

[27] Jones O.G., Decker E.A., McClements D.J. (2010). Comparison of protein–polysaccharide nanoparticle fabrication methods: Impact of biopolymer complexation before or after particle formation. J. Colloid Interface Sci..

[28] Salmon A.R., Parker R.M., Groombridge A.S., Maestro A., Coulston R.J., Hegemann J., Kierfeld J., Scherman O.A., Abell C. (2016). Microcapsule buckling triggered by compression-induced interfacial phase change. Langmuir.

[29] Hirt S., Jones O.G. (2014). Effects of chloride, thiocyanate and sulphate salts on β-lactoglobulin–pectin associative complexes. Intl. J. Food Sci. Technol..

[30] Wu J., Shi M., Wei L., Zhao L., Wang Z., Yan X., Norde W., Li Y. (2015). Pickering emulsions stabilized by whey protein nanoparticles prepared by thermal cross-linking. Colloids Surf. B.

