# Supplementary material for: Impacts of Size and Deformability of β-Lactoglobulin Microgels on the Colloidal Stability and Volatile Flavor Release of Microgel-Stabilized Emulsions
Source: Gels. 2018 Sep 15;4(3):79. doi: 10.3390/gels4030079 (PMC6209270; doi:10.3390/gels4030079)
Supplement: Supplementary file 1 [file gels-04-00079-s001.pdf]

Supplementary Figures – Gels – Murphy and others, 2018

Title: Impacts of size and deformability of β-lactoglobulin microgels on the colloidal stability and volatile flavor release of microgel-stabilized emulsions

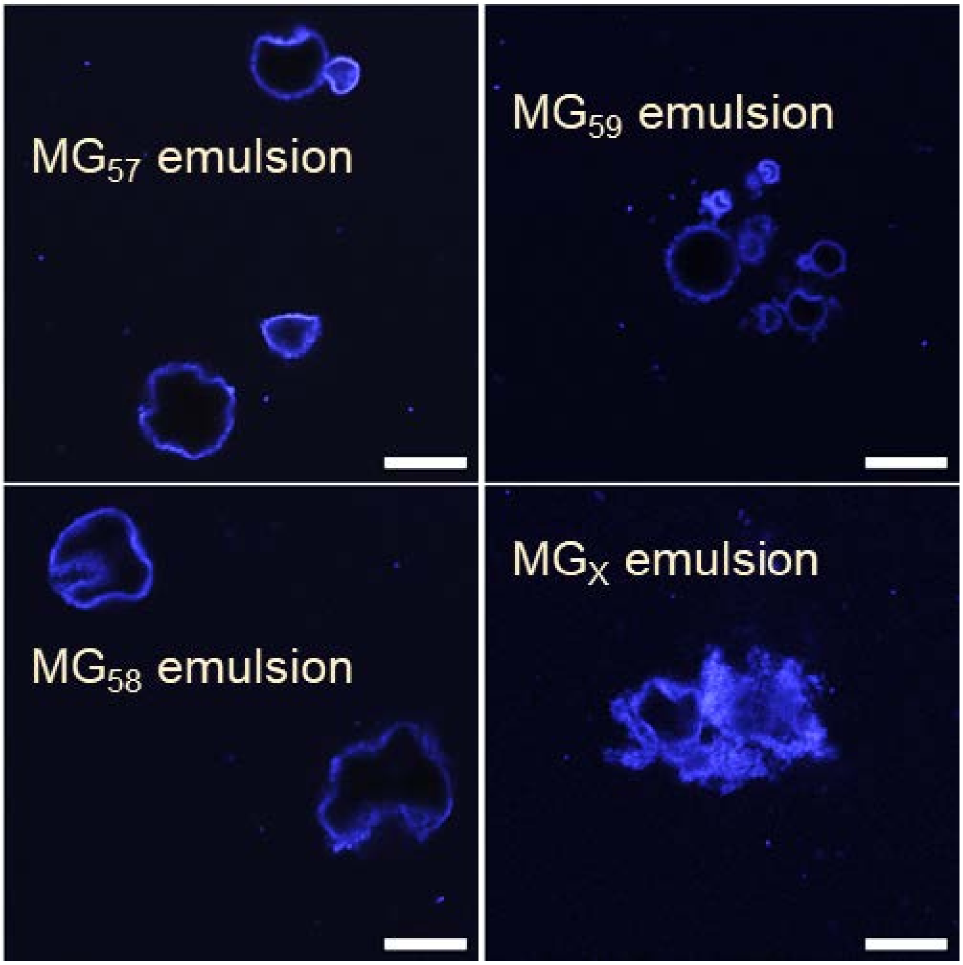

Figure S1: Confocal laser scanning micrographs of limonene/corn oil-in-water emulsions stabilized by Blg microgels after 3 weeks of storage; *scale bars = 20 μm*.

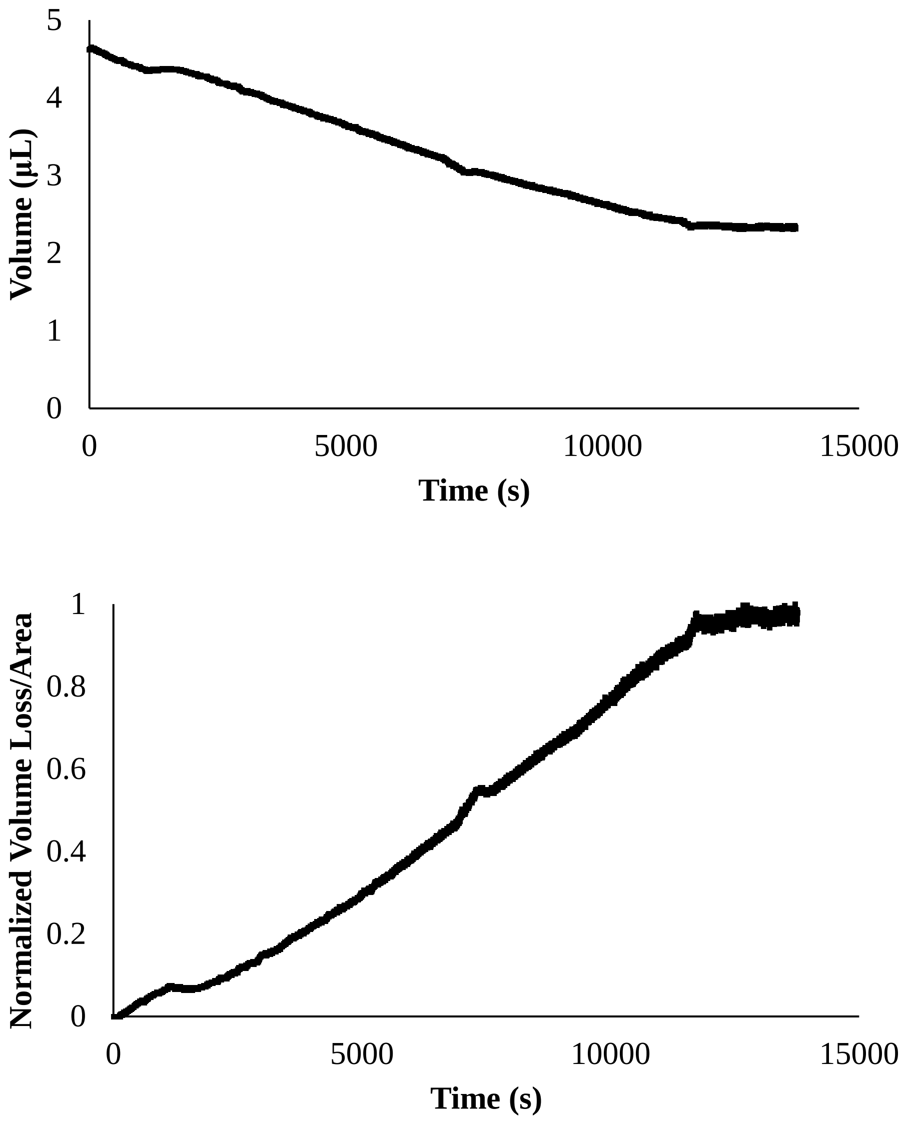

Figure S2: Dynamic volumetric changes in pendant droplets during release of 1-hexanol in MG_{57} suspensions in terms of (A) volume of the pendant droplet or (B) volume loss of pendant droplet normalized by the droplet interfacial area and the total volume loss at long times.
